# Methods for Measuring Autophagy in Mice

**DOI:** 10.3390/cells6020014

**Published:** 2017-06-08

**Authors:** Manon Moulis, Cécile Vindis

**Affiliations:** INSERM, UMR-1048, Institute of Metabolic and Cardiovascular Diseases and University Paul Sabatier, F-31342 Toulouse, France; manon.moulis@inserm.fr

**Keywords:** autophagy, methods, flux, mouse models

## Abstract

Autophagy is a dynamic intracellular process that mediates the degradation of damaged cytoplasmic components by the lysosome. This process plays important roles in maintaining normal cellular homeostasis and energy balance. Measuring autophagy activity is critical and although the determination of autophagic flux in isolated cells is well documented, there is a need to have reliable and quantitative assays to evaluate autophagy in whole organisms. Because mouse models have been precious in establishing the functional significance of autophagy under physiological or pathological conditions, we present in this chapter a compendium of the current available methods to measure autophagy in mice, and discuss their advantages and limitations.

## 1. Introduction

Autophagy is a ‘housekeeping’ subcellular process for lysosome-mediated turnover of damaged proteins and organelles first discovered by Christian De Duve in 1963 [1]. This process is of great importance in maintaining normal cellular homeostasis and energy balance and is found to be ubiquitous in all eukaryotic cells, being highly conserved from yeast to human. Three major forms of autophagy have been described: macroautophagy, microautophagy and chaperone-mediated autophagy. Of these, the most prevalent and common form is macroautophagy, hereafter referred to as autophagy. In this process, the cytoplasmic structures targeted for destruction are sequestered within double-membrane vesicles called autophagosomes and delivered to the lysosome by fusion for breakdown and possible recycling of the resulting macromolecules. The detailed molecular regulation and machinery for autophagy have already been extensively described in several reviews [2]. Briefly, the process of autophagy consists of four sequential steps ending with the degradation of cytosolic “cargo” in lysosomes: initiation and nucleation of phagophore (isolation membrane), expansion of autophagosomes, maturation of autophagosomes into autolysosomes, and execution of autophagy (final degradation). Autophagy is tightly regulated by more than 30 highly conserved genes called ATG (AuTophaGy related genes) that were initially characterized in *Saccharomyces cerevisae* [3,4,5,6] followed by the discovery of their mammalian orthologues [7]. Two major complexes regulate the recruitment of specific proteins into newly forming autophagosomal membranes. One complex involved in the early steps of autophagy comprises ULK1 (also called Atg1) which interacts with Atg13, Atg101 and the focal adhesion kinase family-interacting protein of 200 kDa (FIP200). The other complex requires the class III phosphatidylinositol 3-kinase (PI3K) Vps34 which recruits the autophagy specific proteins Beclin1 (the mammalian orthologue of yeast Atg6), p150/Vps15, Atg14L, or Ambra1 in the region of phagophore formation. The elongation of membranes for the formation of the autophagosome requires two ubiquitin-like conjugating systems. The Atg12-Atg5-Atg16L1 system: Atg12 is conjugated to Atg5 by Atg7 which is similar to an E1 ubiquitin-activating enzyme and Atg10 is similar to an E2 ubiquitin-conjugating enzyme. Then the conjugated Atg12-Atg5 complex interacts with Atg16L1 and associates with phagophores to localize to the outer membrane of nascent autophagosomes, but dissociates before the achievement of autophagosome formation. The second ubiquitin-like reactions involve the microtubule-associated protein 1 light chain 3 (MAP1LC3/Atg8/LC3), the cytosolic form of LC3, LC3-I is generated by the cleavage of pro-LC3 by Atg4 family proteins. LC3-I is then conjugated to the lipid phosphatidylethanolamine (PE) by Atg7 and Atg3 to form LC3-II [8]. Since LC3-II is specifically associated with autophagosomes, the level of LC3-II is correlated with the number of autophagosomes and is considered as an indicator of autophagosome formation [9]. The mature autophagosomes traffic along microtubules using the dynein-dynactin complex, where autophagosomes fuse with endosomes or lysosomes, apparently mediated by endosomal sorting complexes required for transport, soluble *N*-ethylmaleimide-sensitive factor attachment protein receptors (SNAREs), GTPase Rab7 proteins and with the lysosomal-associated membrane proteins, LAMP-1 and LAMP-2. In the final step of the autophagic process, the encapsulated “cargo” is degraded by lysosomal proteases and released back into the cytosol [10].

Mounting evidence supports that autophagy exerts a critical and decisive influence on multiple human physiological and pathophysiological processes, including cancer, neurodegenerative disorders and cardiovascular diseases [11]. Therefore, the pharmacologic modulation of autophagy is increasingly being used in clinical trials, and it is important to determine whether these drugs are truly affecting autophagy, and which step(s) of the process is affected. Hence, there is a growing need among the autophagy community to be able to accurately measure autophagy and to study more particularly its function in vivo. Reliable and quantitative assays to measure autophagy in whole organisms are critical and often challenging. Mouse models for measuring autophagy have been precious in establishing the functional significance of autophagy under physiological or pathological conditions. Consequently, within the past decade numerous models have been developed both to monitor autophagy flux and to modulate autophagy to probe its functions in each cellular process.

We provide in this chapter a compendium of methods to measure autophagy in transgenic and non-transgenic mice using lysosomal blockade. Additionally, we review several methods for inducing autophagy in mice and discuss the advantages and limitations of these methods.

## 2. Monitoring Autophagy Using Transgenic Mice

### 2.1. GFP-LC3 and mCherry-LC3 Mice

A major obstacle to the study of autophagy in vivo is the difficulty of quantifying autophagosomes in tissue. The Mizushima group developed a transgenic mouse in 2004 ubiquitously expressing green fluorescent protein (GFP)-fused LC3 (GFP-LC3) under the constitutive CAG promoter (cytomegalovirus immediate-early (CMVie) enhancer and chicken β-actin promoter) [12]. LC3, the mammalian homologue of yeast Atg8, is present on both the isolation membrane and the completed autophagosomes, thus when GFP-LC3 is expressed, punctate signals are observed by fluorescence microscopy as ring-shaped structures or dots [13,14]. It should be noted that while GFP is a stably folded protein in the cytosol and lumen of early autophagosomes, its fluorescence is quenched by the low pH inside the lysosome and LC3 degradation inside autolysosomes. 

Using this transgenic mouse model, occurrence of autophagy in mouse tissues can be directly monitored by simply creating cryosections and analysis by fluorescence microscopy. The GFP-LC3 transgene does not affect other genes and homozygous mice are healthy and fertile without any abnormal phenotype. Overexpression of GFP-LC3 is found in all tissues except in the brain where the level of GFP-LC3 is comparable to that endogenous LC3 expression suggesting a very rapid turnover of autophagosomes in the brain [15]. Importantly, the overexpression of GFP-LC3 did not affect the endogenous autophagic process and its systematic analysis demonstrated that the regulation of autophagy is organ dependent and its role not restricted to starvation response. However, some caution should be taken when using these mice because GFP-LC3 overexpression can (1) generate protein aggregates and (2) cells possess auto-fluorescent punctate structures such as lipofuscin that is detectable in the green spectra. To avoid these artifacts, GFP-LC3 transgenic mice samples have to be compared with non-transgenic control littermates, with GFP fluorescence detected using only narrow-band pass GFP or FITC filter sets.

Unfortunately, autophagy-positive cardiomyocytes from GFP-LC3 mice cannot be distinguished from other cells such as fibroblasts and smooth muscles in the heart. A cardiac tissue-specific transgenic mouse expressing GFP-LC3 under the control of the α-myosin heavy chain (αMyHC) promoter has been generated [16], but as outlined above, in these mice GFP-LC3 cannot be detected in autolysosomes because of fluorescence quenching and degradative conditions. To circumvent these problems, the Gottlieb group generated transgenic mice expressing mCherry fused to LC3 under the control of the αMyHC promoter (αMyHC-mCherry-LC3) instead of the CAG promoter to detect autophagy only in cardiomyocytes [17]. mCherry is an improved-monomeric red-fluorescent protein, photobleaching resistant which does not lose fluorescence under acidic conditions. Thus, αMyHC-mCherry-LC3 mice allow the detection of both early and late autophagosomes [18]. However, because mCherry proteins remain stable in lysosomes with intact fluorescence, it can lead to an overestimation of the number of autophagosomes. The characterization of the cardiac-targeted mCherry-LC3 mice indicates no apparent effects on cardiac function and endogenous autophagy.

These systemic and tissue-specific models are now successfully used to show reductions in autophagosome numbers in mice deficient in autophagy genes or increases in autophagosome numbers under disease and stress conditions. Of note, cryosections are preferable for preserving fluorescence of the fusion proteins. It is also worth noting that mouse strain can influence interpretation of autophagy analysis, with differences reported using mCherry-LC3 mice in the FVBN or C57BL/6 background [19]. However, the monitoring of GFP-LC3 or mCherry-LC3 dots in transgenic mice is still a convenient method to assess whether different physiological and pathophysiological stimuli regulate autophagosome numbers.

### 2.2. mCherry-GFP-LC3, mRFP-GFP-LC3, and GFP-LC3-RFP-LC3∆G Mice

Static levels of LC3 or scoring of autophagic puncta is an incomplete assessment of autophagy. An increased number of autophagosomes is not always indicative of increased autophagy flux because autophagosomes can accumulate if they are not cleared through lysosomal degradation. Thus, it is necessary to distinguish whether autophagosome accumulation is due to the induction of autophagy or rather a block in autophagosome maturation and degradation, or both.

To overcome these limitations, a transgenic mouse was constructed based on the mRFP-GFP-LC3 reporter, also called the tandem fluorescent-tagged LC3 first developed by the Yoshimori group [18]. Briefly, the principle is that autophagosomes stained by this marker protein showed both mRFP and GFP signals, after the fusion with lysosomes GFP signals were quenched, and only mRFP signals persisted because of its resistance to acidic conditions. A first double-transgenic mouse was generated by cross-breeding cardiac-specific mCherry-LC3 mice with systemic GFP-LC3 mice (αMyHC-mCherry/GFP-LC3 mice) to examine the role of autophagy in the heart [20]. However, this model is limited because the number of mCherry-LC3 and GFP-LC3 molecules are not identical and GFP-LC3 is not cardiac myocyte specific. Hariharan and colleagues circumvented this pitfall by generating a cardiac-specific transgenic mouse harboring tandem fluorescent mRFP-GFP-LC3 fusion protein [21] that has several advantages over the αMyHC-mCherry/GFP-LC3 mice. Since both GFP and mRFP are expressed in a single transgene both green and red fluorescence is emitted from the same LC3 molecule, with 1-1 stoichiometry, thus allowing a more-accurate quantification of autophagosomes and autolysosomes. In addition, the cardiac myocyte-specific manner expression of mRFP-GFP-LC3 in the transgenic mice allows quantification of autophagic flux specifically in cardiac myocytes. This mouse model demonstrated that oxidative stress plays an important role in stimulating autophagic flux, which contributes to myocardial injury during ischemia/reperfusion in vivo [21]. Recently, a systemic mRFP-GFP-LC3 mouse, expressing the tandem fluorescent reporter under the control of the CAG promoter, was developed to study the autophagic flux during renal repair after ischemia-reperfusion injury [22]. In addition to the greatest advantage of maturation step analysis (whether the fusion of autophagosomes with lysosomes is affected or not), mCherry-GFP-LC3 and mRFP-GFP-LC3 have high time resolution. The limitation of the model is in the distinguishment of RFP/GFP-double positive and single positive puncta, technically difficult in vitro and in vivo, dampening accurate measurement of autophagic flux. Futhermore, the measure of basal autophagic flux using this probe is not accurate for puncta detection when there are few autophagosomes.

A novel and simple strategy to deliver and express mCherry-GFP-LC3 reporter in the nervous system was also developed using the intraventricular brain injection of adenovirus-associated vectors (AAVs) in newborn mice. This method results in a wide distribution of the reporter in neurons of the central and peripheral nervous system with high efficiency, allowing the measurement of LC3 flux *in vivo* in single neurons [23]. Recently, a new fluorescent probe GFP-LC3-RFP-LC3∆G was designed by Kaizuka and colleagues to assess autophagy flux in vitro and in vivo [24]. Upon intracellular expression, the probe is cleaved by ATG4 family proteases into equimolar amounts of GFP-LC3 which is degraded by autophagy and RFP-LC3∆G which remains in the cytosol. Autophagic flux can be quantified by the GFP:RFP signal ratio both in cultured cells and in embryos, tissues of zebrafish and mice after eggs probe injection, and systemic transgenic mice [24]. This probe is capable of measuring autophagy flux without using lysosomal inhibitors. In addition it has a strong advantage in the quantification of basal autophagic activity because autophagy flux is quantified as a cumulative index [25]. Important limitations of this new method are that the expression of the probe is significantly different among cells/tissues and time resolution is poor, requiring > 2 h to see a clear reduction of the GFP:RFP ratio, which is significant in terms of monitoring autophagy.

### 2.3. MitoTimer, mt-Keima and Mito-QC Mice

Selective removal of damaged mitochondria through mitophagy is critical for maintaining cellular homeostasis and functions. However, reliable quantitative assays to monitor mitophagy, particularly in vivo, are only just emerging. Three reporters are available to monitor and quantify specifically the mitophagic flux in mouse models: MitoTimer, mt-Keima and mito-QC.

MitoTimer is a time-sensitive fluorescent protein that irreversibly changes its emitted fluorescence from green to red during protein maturation, typically within 48 h after expression. It is fused to the mitochondrial targeting sequence of the COX VIII subunit and therefore localizes to mitochondria. This probe is a useful tool for monitoring real-time mitochondrial aging, turnover and biogenesis, with green, yellow and red mitochondria corresponding respectively to newly synthesized, intermediate and old mitochondria [26]. Hence, to monitor mitochondrial turnover in vivo in the heart, MitoTimer protein has been expressed in mice under the control of the cardiac αMyHC promoter (pC26 αMyHC-MitoTimer) [27]. Interestingly, MitoTimer-positive mitochondria can be isolated and sorted from the heart by flow cytometry for further analysis [27].

Mt-Keima is a pH-dependent fluorescent protein that is resistant to lysosomal proteases and similar to MitoTimer is targeted to mitochondria by fusion to the COX VIII subunit. The mt-Keima probe fluoresces red in acidic pH environment and green when pH is neutral, thus providing a cumulative read-out of autophagic activities [28]. A low ratio of mt-Keima-derived fluorescence (543 nm/458 nm) indicates a neutral environment, whereas a high ratio indicates an acidic pH. The Finkel group has generated a transgenic mouse expressing the mt-Keima reporter for the in vivo assessment of mitophagy in tissues under a wide-range of experimental conditions [29]. Interestingly, they demonstrated tissue-specific differences in the basal levels of mitophagy in mice. However, caution should be taken because when the pH is altered, the Keima protein undergoes a gradual shift in fluorescence excitation, with an overlap in the emission spectra. In addition, the utility of mt-Keima in tissues has some limitations since the Keima protein signal is lost upon conventional fixation, consequently analyses require freshly sectioned tissue and rapid visualization [28].

To circumvent these problems, a new pH-sensitive mitochondrial fluorescent probe was developed, comprised of a functionally inert, tandem mCherry-GFP tag fused to the mitochondrial targeting sequence of the outer mitochondrial membrane protein FIS1 [30]. Under steady-state conditions, the mitochondrial network fluoresces both in red and green, upon mitophagy mitochondria are delivered to lysosomes where mCherry fluorescence remains stable, but GFP fluorescence becomes quenched by the acidic pH. Based on this property, a transgenic mouse model, named mito-QC was generated to monitor mitochondrial turnover and organization in a range of metabolically demanding tissues [30]. The mito-QC mice provide a useful tool for mitophagy assessment in both the developing and mature heart and kidney, and to reveal the diversity of mitochondrial organization in specific subsets of cells within tissues [30]. Compared to MitoTimer and mt-Keima transgenic mice, the mito-QC mice display some advantages such as a full compatibility with a variety of labeling techniques and no overlap in emission spectra [30].

Table 1 summarizes the transgenic mouse models developed to study autophagy and mitophagy flux thanks to fluorescent reporters coupled to LC3 or mitochondrial proteins.

## 3. Measuring Autophagic Flux In Vivo Using Lysosomal Blockade

Autophagic flux is defined as a measure of autophagic degradation activity. A widely-used method consists of a pharmacological blockade acting either by interrupting the autophagosome-lysosome fusion step or by inhibiting lysosome-mediated enzymatic proteolysis [31]. For in vivo studies, current methods are based on mice treated with agents known to block autophagic flux and then monitoring differential accumulation of autophagosomes and increase in LC3-II proteins. Bafilomycin A1, chloroquine and ammonium chloride raise the intracellular pH thus inhibiting autophagosome fusion with the lysosome, nevertheless there remains a debate about whether bafilomycin A1 inhibits autophagosome fusion with lysosome [32]. Bafilomycin A1 is a selective inhibitor of the vacuolar type Na^+^/H^+^-ATPase which disrupts the vesicular proton gradient [33]. Whereas bafilomycin A1 provides an efficient tool for studying autophagic flux in vitro, its use in animals remains costly and unsuitable because bafilomycin A1 needs to be administrated at low doses for short periods or it induces disruption of proteasomal and vesicular dynamics [17]. However, some studies reported its helpful use by the intraperitoneal administration at 0.3 mg/kg/day for 3 days, and showed that autophagy maintains cardiac function during starvation in the adult mice and plays a protective role against cardiomyocytes ischemic death [34,35].

Chloroquine, an anti-inflammatory drug that has been used in the treatment of patients with malaria and inflammatory disorders, is the most commonly used drug in mice to assess autophagic flux because of its suitability in vivo and it is inexpensive compared to bafilomycin A1. The intraperitoneal injection of chloroquine (10–100 mg/kg) allows measurement of autophagic flux in different organs, including the heart [17], the liver [36], the brain as a treatment for Huntington’s disease [37] and in a mouse model of Duchenne muscular dystrophy [38]. The dose of 100 mg/kg has been recently proposed as the most appropriate dose [39] and successfully used in GFP-LC3 mice. Chloroquine was injected 3 h prior to kill the mice for blocking autophagic flux induced by a a rapamycine derivative [40]. Since chloroquine treatment permits comparisons of autophagy flux during the 2–4 h period prior to the end of an experiment, the drug can be injected before, after, or concurrent with the onset of the trial [39].

Colchicine and vinblastine are microtubule-depolymerizing agents that inhibit the autophagosome-lysosome fusion step [41,42]. Studies have shown that intraperitoneal injection at a dose of 0.4 mg/kg/day for colchicine or 2 mg/kg/day of vinblastine for two days, induced the accumulation of the autophagic markers LC3 and SQSTM1/p62 in mouse skeletal muscle [42]. However, Ju and colleagues [43] reported that both LC3-I and LC3-II levels are elevated when mice are treated with colchicine beyond five days suggesting that this upregulation in autophagy could be explained by the compensatory effects of blocking agents used for a long period.

Inhibitors of lysosomal hydrolases and proteases, such as leupeptin, E64d and pepstatin A are also commonly used in cell culture to block autophagic flux [31]. A leupeptin-based assay to characterize the autophagic flux in vivo has been established by intraperitoneal injection of leupeptin at a dose ranging from 9 to 40 mg/kg [36,44]. Using this assay, it has been found that basal autophagic flux in mice measured by LC3b accumulation was greatest in the liver and the retina, while lowest in the spleen. Interestingly, leupeptin pretreatment prior to administering cycloheximide indicated that the LC3b turnover is very rapid with a half-life of 10 to 40 min, depending on the organ studied.

Table 2 summarizes the drugs that can be used in mice to block the degradation of autophagosomes content, by inhibiting fusion with lysosomes or lysosomal enzymatic degradation, and allowing the study of autophagic flux.

## 4. Induction of Autophagy in Mouse Models

### 4.1. By Pharmacological Agents: Rapamycin, Spermidine, Resveratrol and Statins

A well-known approach to activate autophagy is through the modulation of nutrient-sensing signaling pathways. The most commonly targeted component of these pathways is the protein kinase mechanistic Target of Rapamycin (mTOR), which is a potent suppressor of autophagy. Rapamycin, a macrocyclic immunosuppressive agent, has multiple uses such as in immunosuppression, cell proliferation and autophagy stimulation [45]. Rapamycin forms a complex with FK binding protein 12 kDa (FKBP12) in the mechanisticTarget of Rapamycin Complex 1 (mTORC1), and blocks the pro-proliferative signaling pathways by promoting autophosphorylation and dissociation of mTORC1 complex. Intraperitoneal injection of rapamycin (1–10 mg/kg) once a day or 3 times a week for 8 weeks (the half-life of rapamycin is 58–63 h) has been shown to induce increased LC3-II, Beclin1 levels and decreased p62 levels [46]. Besides intraperitoneal injection, rapamycin can also be administered at the dose of 2 mg/kg by oral gavage once a day for 4 weeks [47] or by other routes such as subcutaneously [48]. However, rapamycin treatment-induced in vivo autophagy in mice shows differential effect in heart, muscle and liver [49]. Caution must also be taken because mTOR inhibition is not a specific inducer of autophagy and short versus long-term rapamycin treatment may affect a wide range of cellular responses, particularly protein synthesis and cellular metabolism, in addition to autophagy activation. Comparing the effects of injecting adult male mice with rapamycin for 2, 6, or 20 weeks, Fang and colleagues [50] demonstrated that mice experienced negative effects of rapamycin treatment, including insulin resistance with short duration, but insulin signaling changed from an insulin resistant to an insulin-sensitive state after 20 weeks of rapamycin treatment. Recently, it has been shown that the short-term administration of the mTORC1 inhibitor everolimus (a rapamycine derivative) inhibits the phosphorylation of the mTORC1 substrates S6rp and ULK1 resulting in robust induction of autophagy in mice. Using osmotic minipumps, GFP-LC3 transgenic mice were treated continuously either with vehicle or everolimus (1.5 mg/kg per day) for 3 or 28 days. Alternatively, a regimen consisting of intermittent everolimus administration (every other day) for 56 days by oral gavage have been tested. However, continuous long-term administration of everolimus triggers adaptation mechanisms resulting in hyperphosphorylation of ULK1 and inhibition of autophagy. Long-term administration of everolimus intermittently rescues responsiveness to the drug only in the most sensitive substrate (S6rp) without any effect on autophagy [40]. In addition, adverse effects such as dyslipidemia and hyperglycemia have recently been identified in mice receiving everolimus (1 or 5 mg/kg) for 12 weeks [51].

Alternative, nontoxic autophagy inducers such as resveratrol and spermidine, are also being evaluated for their potential to induce autophagy in vivo [52,53]. Resveratrol is a natural polyphenol found in grapes, red wine or berries, and has been suggested to mediate the cardioprotective effects of red wine. Resveratrol is a potent inducer of autophagy [54], and this effect is mediated through the activation of sirtuin1 (SIRT1), a NAD+-dependent deacetylase [55]. Intraperitoneally injected optimal dose of resveratrol (25 mg/kg) into GFP-LC3 transgenic mice 3 h before sacrifice, induces autophagy in an array of organs as shown by increased GFP-LC3 dots in heart, liver and muscle tissue sections [53]. Alternatively, rodents could be fed with a diet enriched with resveratrol with short-term/low dose [56] versus long-term/high dose treatment [57], and autophagy induction was shown by increased levels of LC3-II, Beclin1 and TEM images of autophagic vacuoles. In addition to act as an autophagy inducer, resveratrol exhibits antioxidant effects such as a down-regulation of redox genes and an up-regulation of antioxidant enzymes which contribute to its health benefits [58].

Spermidine is a polyamine with high content in citrus fruit, dry soy bean, chicken liver, green peas, corn, shell fish and blue cheese which [59] has been shown to increase the lifespan of yeast, nematodes, and flies in an autophagy-dependent fashion [52] especially in reducing age-related oxidative protein damage. Dietary spermidine elicits cardioprotective effects in aged mice through enhancing cardiac autophagy and mitophagy [60]. Spermidine could be either orally administered in drinking water (3 to 30 mM) that was replenished or replaced every 2–3 days, or intraperitoneally injected at the dose of 50 mg/kg [53,61,62]. As shown recently by Eisenberg and colleagues [63], mice supplemented with spermidine have to be treated with leupeptin for the final 4 weeks to allow basal autophagic flux assessment. The capacity of orally supplemented spermidine to induce autophagy or mitophagy flux in vivo was corroborated using transgenic cardiomyocyte-specific tandem-fluorescent mRFP-GFP-LC3 mice under chloroquine treatment and in mt-Keima mice [63]. Notably, unlike other longevity promoting agents, spermidine had no detectable effects on glucose and insulin metabolism.

Finally, statins, a family of drugs widely used as inhibitors of cholesterol biosynthesis by acting as competitive inhibitors of HMG-CoA reductase, a rate-limiting enzyme of the cholesterol biosynthesis pathway, had been recently described to enhance autophagy. Statins were used in mice intraperitoneally injected or formulated in the drinking water and food [64,65]. For example, hepatocytes of mice fed with statin for 16 weeks showed prominent vacuolization and autophagosomes, as assessed by TEM analysis [66]. Intraperitoneal daily injection of 20 mg/kg simvastatin for 12 weeks induces an increase in autophagy-related proteins Atg5, LC3b and Beclin1 expression and autophagosome formation [67].

It should be noted that resveratrol and spermidine stimulate autophagy through mTOR-independent or -dependent mechanisms. Resveratrol has been shown to stimulate autophagy through the activation of the deacetylase SIRT1 [53], but could also directly inhibit mTOR kinase activity through ATP competition (Park 2016 Scientific reports) whereas spermidine is thought to act as an inhibitor of acetylases [52]. The observed autophagic response induces by statins seems associated with the reduction of phosphorylated Akt levels accompanied by a decrease in the activation of mTOR and its substrate ribosomal p70S6 kinase [68].

### 4.2. Under Physiopathological Conditions: Starvation, Exercise and Hypoxia

The most potent known physiological inducer of autophagy is starvation. It is the most rapid and easiest method for stimulating the induction of the autophagy machinery in mice. Mizushima and colleagues measured an autophagy response in mice deprived of food for 24 or 48 h [12]. During starvation experiments, mice should have water ad libitum and their temperature and blood pressure checked periodically. Although autophagy is regulated differently among organs, autophagy is still induced in most organs in response to nutrient starvation. However, the appropriate time of starvation should be determined empirically because autophagic responses to various stimuli or in disease states can differ between organs [69]. A strong induction of autophagy has been observed in the liver, heart, and skeletal muscle of fasting mice [12,70,71]. Recently, Pietrocola and colleagues showed that in circulating leukocytes from mice that underwent 48 h of fasting the increase in LC3b was only detectable following leupeptin injection 2h before drawing blood [72]. However, it has been shown that LC3b expression fluctuated excessively among individual mice, implying that autophagic induction was not synchronized in the mice examined. To synchronize autophagic induction in all mice of an experimental group, Ezaki and colleagues conducted a starvation/feeding/re-starvation regimen [70] where mice are fasted for 24 h, then fed for 2 h in the dark to suppress autophagy to a minimal level and re-fasted to induce autophagy. Hence, synchronizing autophagic induction in all mice facilitated the statistical analysis of the experimental data. In addition, mice are nocturnal animals that prefer to move and eat during the night, thus for short-term starvation it is recommended to fast mice at night, while for long-term starvation it is better to start food deprivation early in the morning to avoid the possibility that the animals have already been fasting for several hours [69].

Autophagy can also be induced by other physiopathological stresses such as exercise or hypoxia. Activation of autophagy has been well documented in response to both acute and chronic endurance exercise, the first study that observed autophagic vacuoles during exercise dates back to 1984 [73]. Recently, tissues of GFP-LC3 mice were analyzed after treadmill exercise, and in both skeletal and cardiac muscle GFP-LC3 puncta numbers increased while degradation of SQSTM1/p62 was observed after 30 min of running and became stabilized at 80 min [74]. Tonic, oxidative muscles have higher autophagic flux and mitophagy protein expression than phasic, glycolytic muscles [75]. Additionally, it has been shown that exercise induced autophagic flux in other organs involved in glucose and energy homeostasis, such as liver and pancreas, and also islet β-cells and adipose tissue [74]. Therefore, when measuring autophagy flux induced by exercise, caution must be taken because a larger activation of autophagy is observed when exercise is performed in a fasted state compared with a fed state [76].

Several studies have reported increased autophagic vacuole formation or altered autophagic activity during ischemia-reperfusion (I/R), or chronic ischemia. Indeed, the consecutive hypoxic and oxidative stress evoked by I/R has been shown to enhance autophagy in different rodent models. Cardiac I/R models have been the more described model in mice [77,78], other models, such as brain [79], renal [80] or hepatic [81] I/R have been also studied. Following mild ischemia (25–40 min) by artery ligation autophagy is upregulated and showed protective properties, whereas detrimental effects have been observed when the period of ischemia is prolonged to 40–60 min or during the subsequent reperfusion phase [22]. The extent of autophagic flux after I/R has been generally evaluated most conveniently using transgenic mouse models expressing tagged-LC3 (GFP-LC3, mCherry-LC3 or mRFP-GFP-LC3) that have been described above.

Table 3 summarizes the compounds and conditions that can be used in mice to induce autophagy more or less specifically or physiologically, and allowing to study its function in diverse physiological and pathophysiological contexts.

## 5. Conclusions

Mouse models developed for measuring autophagy in vivo offer many advantages: observation of the distribution and the size of autophagosomes, quick analysis of many tissues and cells, and the real-time observation of autophagy in living cells. However, some technical limitations should be considered when autophagic flux is measured in mice because of the variability between animals that do not always induce autophagy at the same time. To reduce variability and to improve the statistical relevance, a *calculation* of *sample size* by *power* analysis is one of the most important components for the design of animal studies. In addition, basal autophagy or sensitivity to autophagy induction may be impacted by mouse age, sex or strain background. Because mice are nocturnal animals, appropriate caution is also required when designing fasted-mice experiments to avoid circadian effects, and for the same reason replicate experiments should be conducted at the same time of day. Importantly, it has been reported that the bioavailability of autophagy-inducing and inhibiting drugs is likely tissue specific thus implying that these methods need to be optimized for each tissue analysis. In summary, mouse models for monitoring autophagy are valuable tools for establishing that autophagy plays an important role in cellular homeostasis, and to study its function in diverse pathophysiological contexts, particularly in the pathophysiology of disease.

## Figures and Tables

**Table 1:** Transgenic mouse models for monitoring autophagy.

Transgenes/Probes	Tissues	Processing	Techniques	Analyses	Limitations/Advantages
**GFP-LC3** (systemic and cardiac-specific models)	Heart, Liver, Muscle, Pancreas, Kidney, Brain				No autophagic flux
**mCherry-LC3** (cardiac-specific model)	Heart	Protein extraction	Western blot	LC3 and GFP-LC3 expression and lipidation	
**mCherry-GFP-LC3** (cardiac-specific model)	Heart				Restricted to the cardiac tissue/Autophagic flux (but not in basal)
**mRFP-GFP-LC3** (systemic and cardiac-specific models)	Heart, Kidney	Cryosections	Fluorescence and electron microscopy	Red or green LC3 puncta expression, autophagosomes number, area and GFP:RFP ratio	
**GFP-LC3-RFP-LC3∆G** (systemic model)	Embryos, Muscle				Autophagic flux (included in basal) and no lysosomal inhibitors need
**MitoTimer** (cardiac-specific model)	Heart			Tracking of red and green channels for mitochondrial “aging” or flux	Mitophagic flux, mito-QC compatible with fixation and no fluorescence spectrum overlap
**mt-Keima** (systemic model)	Heart, Brain, Liver, Thymus	Cryosections	Fluorescence microscopy		
**mito-QC** (systemic model)	Heart, Brain, Muscle, Liver, Spleen, Kidney	Mitochondria isolation	Flux cytometry		
**mCherry-GFP-LC3** (injection and AAVs delivery in new born mouse)	Nervous system	Cryosections	Fluorescence microscopy	Red or green LC3 puncta expression	Restricted to the nervous system/Autophagic flux, wide distribution
**GFP-LC3-RFP-LC3∆G** (injection in mouse embryo)	Embryos, Muscle	Cryosections	Fluorescence microscopy	GFP:RFP ratio	Differential tissue expression and poor time resolution

**Table 2:** Drugs targeting lysosomal blockade for measuring autophagy flux in mice.

Drugs	Comments	Administration	Doses	Limitations/Advantages
**Leupeptin**	Cystein, serine, threonine proteases inhibitor	Intraperitoneally	9–40 mg/kg	Most commonly used in vivo
**E64d**	Cystein proteases inhibitor	Orally (food)		Preferentially used in vitro
**Pepstatin A**	Aspartyl proteases inhibitor *Lysosomal protein degradation blockage*	Intraperitoneally	20 mg/kg	Should or can be used in combination
**Bafilomycin A1**	Na+/H+-ATPase inhibitor	Intraperitoneally	0.1–1mg/kg	Costly and unsuitable in vivo
**Chloroquine**		Intraperitoneally	10–100 mg/kg	Quite inexpensive and suitable in vivo, most commonly used
**Ammonium chloride (NH4Cl)**		Orally (drinking water)		Less frequent
	*Autophagosome-lysosome fusion blockage*			
**Colchicine**	Microtubules depolymerizing agents	Intraperitoneally	0.4–2 mg/kg	Lack of specificity, clastogenic effects
**Vinblastine**				
	*Autophagosome-lysosome fusion blockage*			

**Table 3:** Selected autophagy inducers for measuring autophagy flux in mice.

Drugs or Conditions	Comments	Administration	Doses/Time	Limitations/Advantages
**Rapamycin**	mTOR inhibitor	Intraperitoneally	1–10 mg/kg (daily or several times per week for several weeks)	Lack of specificity, partial autophagy induction
**Resveratrol**	Natural polyphenol	Intraperitoneally or orally (food or drinking water)	25 mg/kg	Lack of specificity, non toxic
**Spermidine**	Polyamine		50 mg/kg	
**Statins**	Cholesterol biosynthesis inhibitors		20 mg/kg (daily for several weeks)	
**Starvation**		Food deprivation with water ad libitum	12–48 h	The most rapid and easiest method, wide induction
**Exercise**		Treadmill running	60–90 min at ~10 m/min	Difficult to standardize, multifactorial
**Hypoxia**		Artery ligation	25–40 min	Invasive, detrimental effects if prolonged
